# ﻿Pezizomycotina species associated with rotten plant materials in Guizhou Province, China

**DOI:** 10.3897/mycokeys.106.125920

**Published:** 2024-06-27

**Authors:** Shamin Fu, Jing-E Sun, Entaj Tarafder, Nalin N. Wijayawardene, Yan Hu, Yong Wang, Yan Li

**Affiliations:** 1 The Key Laboratory of Plant Resources Conservation and Germplasm Innovation in Mountainous Region (Ministry of Education), College of Life Sciences/Institute of Agro-Bioengineering, Guizhou University, Guiyang 550025, China; 2 College of Agriculture, Guizhou University, Guiyang Guizhou 550025, China; 3 Guizhou Zhunongjia Agricultural Science and Technology Service Co., Ltd, Guiyang, Guizhou 550025, China; 4 Institute of Plant Health and Medicine, College of Agriculture, Guizhou University, Guiyang Guizhou 550025, China; 5 Center for Yunnan Plateau Biological Resources Protection and Utilization, College of Biological Resource and Food Engineering, Qujing Normal University, Qujing, Yunnan 655011, China; 6 Weining Branch, Bijie Tobacco Company, Bijie, 553100, China

**Keywords:** Ascomycota, morphology, phylogeny, taxonomy, three new taxa

## Abstract

Nine Pezizomycotina strains were isolated from rotten dead branches and leaves collected from Guizhou Province. To obtain their accurate taxonomic placement, we provided the morphological characteristics of conidiophore cells and conidia. Phylogenetic relationships, based on ITS, *rpb2*, SSU, LSU and *tub2* gene sequences, confirmed our strains represented three novel species, *Peglioniafalcata*, *Neoascochytapseudofusiformis* and *Neomicrosphaeropsiscylindrica*. *Peglioniafalcata* produced falcate conidia and *Neoa.pseudofusiformis* generated fusiform conidia, while *Neom.cylindrica* possessed cylindrical conidia. The phylogenetic results also supported them as novel taxa. All the new species in the present study were found as saprophytic on forest litter with high rainfall, which suggest they may have a certain effect on nutrient decomposition and redistribution in forest ecosystems. Thus, it opened a way for further research on related ecological roles and their application production.

## ﻿Introduction

Pezizomycotina is the largest subphylum of Ascomycota (Spatafora et al. 2006; Wijayawardene et al. 2022a), which is large and diverse (the 10^th^ edition of Ainsworth & Bisby’s Dictionary of the Fungi estimates close to 70,000 Pezizomycetes species) (Charley and David 2017). These taxa often exist as saprophytes fed by herbivore faeces or grow on woody and non-woody plant tissues. However, some can also be pathogenic to some plants and animals or symbiotic with some plants as endophytes (Money 2016). Fungal studies, related to ascomycetous taxa have been extensively carried out in China and many novel taxa are introduced annually (Hyde et al. 2016; Wijayawardene et al. 2022b).

The genus *Peglionia* (Goidànich 1934) is distinguished from *Circinotrichum* and *Gyrothrix* by the branching pattern of the setae (Hernández-Restrepo et al. 2022). According to Hernández-Restrepo et al., *Gyrothrixverticiclada* (CBS 127654 phenotype, CBS 140226 and CBS 148329) clustered with *G.hughesii* in the phylogenetic tree as a distinct branch, which were defined as a new genus (*Peglionia*) (Hernández-Restrepo et al. 2022). *Peglioniaverticiclada* (type species) is characterised by the production of curved conidia and setae with verticillate and straight branches at the apex and never circinate as in *Circinotrichum*, *Gyrothrix* or other members of Xylariales. In terms of morphological characteristics, the conidiogenous cells of *Peglionia* vary from inverted clavate to lagenate, appearing hyaline or subhyaline. The conidia are attached to the tips of the conidiogenous cells, presenting a dry sickle shape, without septa and presenting hyaline (Hernández-Restrepo et al. 2022).

*Neomicrosphaeropsis* (Didymellaceae) was introduced by Thambugala et al. (2017) based on morphology and molecular data; meanwhile, *Phomatamaricicola* were recollected and accommodated to *Neomicrosphaeropsis*. There are currently 11 epithets in the genus *Neomicrosphaeropsis* in Mycobank (www.mycobank.org), but *Neom.cystisi*, *Neom.cystisicola*, *Neom.cytisina* and *Neom.minima* have been transferred to *Microsphaeropsis* (Wijayawardene et al. 2022b). This genus contains some pathogens or endophytes (Wijayawardene et al. 2017). In terms of morphological features, the conidiophores of the genus *Neomicrosphaeropsis* appear pustulate, almost submerged in agar and slightly vesicular. The outer wall of the conidiophore ranges from light to dark brown. Conidiogenous cells are cylindrical, appear hyaline, have a smooth surface and are aggregated singly or in multiples. Conidia are hyaline to light brown, with a smooth outer wall and are ovate to ellipsoid (Thambugala et al. 2017).

Chen et al. (2015) introduced *Neoascochyta* to accommodate taxa that morphologically resemble to *Ascochyta*, but phylogenetically distinct. *Neoascochyta* belongs to the Didymellaceae family and species in this family are primarily parasitic on wood and dead herbaceous stems or leaves (Hyde et al. 2013). Currently, 20 *Neoascochyta* species have been listed in MycoBank database (2024). This genus is morphologically characterised by pseudothecial ascomata, cylindrical to subclavate asci, cylindrical to ovoid, hyaline 1-septate ascospores. The asexual morph is coelomycetous and is characterised by pycnidial conidiomata, pseudoparenchymatous wall, obpyriform or ampulliform to doliiform conidiogeneous cells and hyaline fusoid to cylindrical, obclavate-ovoid to ellipsoidal conidia (Chen et al. 2015).

The purpose of this study was to introduce three new Pezizomycotina taxa collected in Guizhou Province, viz. *Peglioniafalcata*, *Neoascochytapseudofusiformis* and *Neomicrosphaeropsiscylindrica*. The present study was of great significance to enrich the diversity of Pezizomycotina in southwest China on the basis of morphological description and phylogeny combined with ITS, LSU, *tub2* and *rpb2* sequence data analysis. Meanwhile, since all three new species identified are saprophytic fungi, which play an important role in the process of organic matter decomposition, they can be further studied for their ecological effects, which will provide an important theoretical and practical basis for relevant applied research and potential value exploration, based on their roles in natural ecosystems.

## ﻿Materials and methods

### ﻿Fungal sampling, isolating and morphology

Sample collection was carried out in the summer of 2023, in a mountain forest in Yunyan District of Guiyang City, Guizhou Province, which was at a time of high rainy weather, with a large area covered by various kinds of vegetation. Decayed plant tissue samples were collected from the moist soil surface and brought back to the laboratory in self-sealing bags. The specimens were then examined for their macroscopic characteristics using a Nikon SMZ 745 series stereomicroscope and photographed, using a Canon 700D digital camera. Pure cultures were obtained using a single spore isolation method as described in (Senanayake et al. 2020). The germinated spores were transferred to fresh potato dextrose agar (PDA) plates and incubated at 25 °C for 14 days. Micro-morphological structures were photographed using a Nikon digital camera (Canon 700D) that was attached to a light microscope (Nikon Ni). Fruiting bodies on natural substrates were observed using a Zeiss Scope5 compound microscope Axioscope 5 (Carl Zeiss Microscopy GmbH, Jena, Germany) with the microscope techniques of differential interference contrast light (DIC) and photographed using an AxioCam 208 colour (Carl Zeiss Microscopy GmbH, Jena, Germany) camera and saved as JPG files. Approximately 30 morphological measurements of new species were made of each feature using the ZEN 3.0 (blue edition) (Jena, Germany) software.

Type specimens were deposited in the Herbarium of the Department of Plant Pathology, Agricultural College, Guizhou University (**HGUP**). Ex-type cultures were deposited in the Culture Collection at the Department of Plant Pathology, Agriculture College, Guizhou University, P.R. China (**GUCC**). Taxonomic information of the new species was submitted to MycoBank (www.mycobank.org) and accession numbers are provided in the Taxonomy section of this paper.

### ﻿DNA extraction, polymerase chain reaction (PCR) amplification

The fungal strains were cultured on potato dextrose agar (**PDA**) (c = 40.1 g/l) medium in an incubator at 25 °C for 7 days and the mycelium was scraped with a sterile scalpel. Total DNA was extracted with a (Biomiga#GD2416, San Diego, California, USA) BIOMIGA Fungus Genomic DNA Extraction Kit (GD2416) following the manufacturer’s protocol. Five loci (ITS, *tub2*, SSU, LSU and *rpb2*) were selected for the total DNA extracted. Amplification was undertaken of forward and reverse primers, including the internal transcribed spacer regions (**ITS**), partial beta-tubulin gene (*tub2*), partial large subunit nrRNA gene (**LSU**), 18S small subunit ribosomal RNA (**SSU**) and partial DNA-directed RNA polymerase II second largest subunit (*rpb2*) gene using the primer pairs ITS5/ITS4 (White et al. 1990), Bt2a/Bt2b (Woudenberg et al. 2009), LR0R/LR5 (Vilgalys and Hester 1990), NS1/NS4 (White et al. 1990) and RPB2-5F2/RPB2-7cR (Liu et al. 1999), respectively. Amplification reactions profiles for LSU, ITS, and *tub2* gene followed to Chen et al. (2015) and SSU accorded to White et al. (1990). The amplification for *rpb*2 was performed according to an improved protocol (Crous et al. 2013). PCR products were sequenced by SinoGegoMax (Beijing, China). The consensus sequences were assembled from forward and reverse sequences using Seqman Pro v. 10.0.1 (DNASTAR, Madison, USA). Novel sequences generated in this study were deposited in GenBank (http://www.ncbi.nlm.nih.gov) and their accession numbers are shown in Table 1.

**Table 1.** Taxa and corresponding GenBank accession numbers of sequences used in the phylogenetic analysis T = ex-holotype strain, F = non-type strain, ET = ex-epitype strain.

Current name	Old name	Strain number	T/F	Host	Country	GenBank Accession Numbers				
						ITS	LSU	*tub2*	*rpb2*	SSU
*Circinotrichumcircinatum*	“*Gyrothrixcircinata*”	CBS 140217	F	Unidentified	Malawi	ON400747	ON400800	−	ON399330	−
	“*Gyrothrixcircinata*”	CBS 140218	F	Unidentified	Malawi	ON400748	ON400801	−	ON399331	−
	“*Gyrothrixcircinata*”	CBS 140229	F	Unidentified	Zimbabwe	ON400751	ON400804	−	ON399335	−
	“*Gyrothrixcircinata*”	CBS 140230	F	Unidentified	Zimbabwe	ON400752	ON400805	−	ON399334	−
	“*Gyrothrixcircinata*”	CBS 140219	F	Unidentified	Malawi	ON400749	ON400802	−	ON399332	−
	“*Gyrothrixcircinata*”	CBS 140220	F	Unidentified	Malawi	ON400750	ON400803	−	ON399333	−
	“*Gyrothrixcircinata*”	CBS 148325	F	Unidentified	USA	ON400745	ON400798	−	ON399329	−
	“*Gyrothrix* sp.”	CBS 140235	F	Unidentified	Brazil	ON400746	ON400799	−	ON399336	−
	“*Gyrothrixcircinata*”	CBS 148326	F	Unidentified	Australia	ON400743	ON400796	−	ON399328	−
	“*Gyrothrixcircinata*”	CBS 148327	F	*Hakea* sp.	Australia	ON400744	ON400797	−	ON399327	−
	“*Gyrothrix* sp.”	CPC 26309	F	*Erica* sp.	France	ON400742	ON400795	−	ON399326	−
*Circinotrichummaculiforme*	*Circinotrichummaculiforme*	CBS 122758	F	Unidentified	Spain	KR611875.1	KR611896.1	−	ON399337	−
	*Circinotrichummaculiforme*	CBS 140016	ET	*Loranthus* sp.	Czech Republic	KR611874.1	KR611895.1	−	ON399338	−
	*Circinotrichummaculiforme*	CBS 140225	F	Unidentified	Cuba	ON400753	ON400806	−	ON399339	−
	*Circinotrichum* sp.	CPC 29975	F	*Cornussanguinea*	France	ON400754	ON400807	−	ON399340	−
“*Ceratocladiummicrospermum*”	“*Ceratocladiummicrospermum*”	CBS 488.77	F	*Quercus* sp.	Slovakia	ON400740	ON400793	−	ON399324	−
*Circiontrichumaustraliense*	“*Gyrothrixpodosperma*”	CBS 148706	T	Unidentified	Australia	ON400741	ON400794	−	ON399325	−
	*Coniocessianodulisporioides*	CBS 125776	F	Unidentified	Unknown	MH863754.1	MH875222.1	−	−	−
	*Coniocessianodulisporioides*	CBS 125777	F	Unidentified	Unknown	MH863755.1	MH875223.1	−	−	−
*Coniocessiacruciformis*	*Coniocessiacruciformis*	CBS 125769	F	*Triticumaestivum*	Iran	MH863750.1	MH875218.1	−	−	−
*Pirozynkiomycesbrasiliensis*	“*Gyrothrixcircinata*”	CBS 112314	T	Unidentified	Brazil	ON400767	ON400819	−	ON399341	−
*Circinotrichumsinense*	*Circinotrichumsinense*	UAMH 11913	T	*Camelliacuspidat*a	China	KY994106.1	KY994107.1	−	−	−
*Hansfordiapruni*	*Hansfordiapruni*	CBS 125775	F	*Prunuspersica*	Italy	MH863753.1	MH875221.1	−	−	−
	*Hansfordiapruni*	CBS 125767	F	*Prunuspersica*	Italy	MH863748.1	MH875216.1	−	−	−
	*Hansfordiapruni*	CBS 125768	F	*Prunuspersica*	Italy	MH863749.1	MH875217.1	−	−	−
	*Selenodriellafertilis*	CBS 772.83	F	Unidentified	Unknown	KP859055.1	KP858992.1	−	−	−
	*Selenodriellafertilis*	CPC 16273	F	Unidentified	Unknown	ON400771	ON400823	−	ON399358	−
	*Selenodriellafertilis*	CBS 144589	F	Unidentified	Unknown	MK442624.1	MK442560.1	−	−	−
*Circinotrichumrigidum*	“*Circinotrichumrigidum*”	CBS 148328	F	*Eucalyptus* sp.	Australia	ON400772	ON400824	−	ON399359	−
*Selenodriellabrasiliana*	“*Circinotrichumaustraliense*”	CBS 140227	T	Unidentified	Brazil	ON400769	ON400821	−	ON399356	−
	“*Circinotrichum* sp.”	CBS 140236	F	Unidentified	Brazil	ON400770	ON400822	−	ON399357	−
*Selenodriellacubensis*	*Selenodriellacubensis*	CBS 683.96	T	Unidentified	Cuba	KP859053.1	KP858990.1	−	−	−
*Peglioniaverticiclada*	“*Gyrothrixverticiclada*”	CBS 101171	F	Unidentified	Venezuela	ON400766	ON400818	−	ON399355	−
	“*Gyrothrixverticiclada*”	CBS 140226	F	Unidentified	Venezuela	ON400764	ON400816	−	ON399354	−
	“*Gyrothrixverticiclada*”	CBS 127654	ET	*Smilaxaspera*	Italy	ON400763	ON400815	−	ON399352	−
	“*Gyrothrixverticiclada*”	CBS 148329	F	*Eucalyptus* sp.	Australia	ON400765	ON400817	−	ON399353	−
	***Peglioniafalcata***	**GUCC 23-0042**	**T**	**Unidentified**	**China**	**PP295269**	**PP314032**	−	**PP396044**	−
	***Peglioniafalcata***	**GUCC 23-0043**	**F**	**Unidentified**	**China**	**PP295270**	**PP314033**	−	**PP396045**	−
	***Peglioniafalcata***	**GUCC 23-0044**	**F**	**Unidentified**	**China**	**PP295271**	**PP349828**	−	**PP396046**	−
	*Microdochiumlycopodinum*	CBS 125585	F	Unidentified	Unknown	NR_145223.1	KP858952.1	−	KP859125.1	−
	*Idriellalunata*	CBS 204.56	F	*Fragaria chiloensis var. ananass*a	USA	MH857584.1	MH869129.1	−	−	−
	*Zygosporiumpseudomassoni*	CBS 146059	F	Unidentified	Unknown	NR_166342.1	NG_068340.1	−	MN556815.1	−
	*Zygosporiummycophilum*	CBS 894.69	F	Unidentified	Unknown	MH859474.1	MH871255.1	−	−	−
	*Monosporascuscannonballus*	ATCC 26931	T	*Cucumismelo*	USA	NR_111370.1	−	−	−	−
	*Monosporascusnordestinus*	CMM 4846	F	*Trianthemaportulacastrum*	Brazil	MG735241	MG748810.1	−	−	−
	*Monosporascuscaatingaensis*	CMM 4833	F	*Boerhaviadiffusa*	Brazil	MG735228.1	MG748797.1	−	−	−
	*Diatrypellavulgaris*	CBS 128329	F	*Citrusparadisi*	Australia	MH864880.1	MH876328.1	−	−	−
	*Diatrypedisciformis*	CBS 197.49	F	Unidentified	Unknown	−	DQ470964.1	−	DQ470915.1	−
	*Acrocordiellaocculta*	CBS 140500	F	Unidentified	Unknown	KT949893.1	MH878156.1	−	−	−
	*Neomicrosphaeropsisalhagi-pseudalhagi*	MFLUCC 17-0825	T	*Alhagipseudalhagi*	Uzbekistan	MH069664	MH069670	MH069689	−	MH069676
	*Neomicrosphaeropsiselaeagni*	MFLUCC 17-0740	T	*Elaeagnusangustifolia*	Russia	MH069666	MH069672	MH069691	−	MH069678
	*Neomicrosphaeropsisitalica*	MFLUCC 15-0485	T	*Tamarix* sp.	Italy	KU900318	KU729854	−	−	KU900309
	*Neomicrosphaeropsisitalica*	MFLUCC 16-0284	F	*Tamarix* sp.	Italy	KU900321	KU900296	KX453299	−	KU900311
	*Neomicrosphaeropsisitalica*	MFLUCC 15-0484	F	*Tamarix* sp.	Italy	KU900319	KU729853	KX453298	−	−
	*Neomicrosphaeropsisitalica*	MFLUCC 15-0487	F	*Tamarix* sp.	Italy	KU900320	KU729852	−	−	KU900310
	*Neomicrosphaeropsisjuglandis*	MFLUCC 18-0795	T	*Juglansregia*	Turkey	MN244223	MN244206	MN871954	−	MN244183
	*Neomicrosphaeropsisnovorossica*	MFLUCC 14-0578	T	*Tamarixramosissima*	South European Russia	KX198709	KX198710	−	−	KX198711
	*Neomicrosphaeropsisrossica*	MFLUCC 14-0586	T	*Tamarixramosissima*	South European Russia	KU752192	KU729855	−	−	KU870914
	*Neomicrosphaeropsistamaricicola*	MFLUCC 14-0443	F	*Tamarix* sp.	Italy	KU900322	KU729851	−	−	KU900312
	*Neomicrosphaeropsistamaricicola*	MFLUCC 14-0439	F	*Tamarix* sp.	Italy	KU900323	KU729858	−	−	KU900313
	*Neomicrosphaeropsistamaricicola*	MFLUCC 14-0602	T	*Tamarix* sp.	Italy	KM408753	KM408754	MH069692	−	KM408755
	***Neomicrosphaeropsiscylindrica***	**GUCC23-0048**	**T**	**Unidentified**	**China**	**PP314028**	**PP314039**	**PP396056**	**PP396050**	**PP316087**
	***Neomicrosphaeropsiscylindrica***	**GUCC23-0049**	**F**	**Unidentified**	**China**	**PP314030**	**PP316086**	**PP396057**	**PP396051**	**PP316089**
	***Neomicrosphaeropsiscylindrica***	**GUCC23-0050**	**F**	**Unidentified**	**China**	**PP314031**	**PP316082**	**PP396058**	**PP396052**	**PP316088**
*Microsphaeropsisminima*	*Neomicrosphaeropsisminima*	MFLUCC 13-0394	F	*Verbascum* sp.	Italy	KX572336	KX572341	−	−	KX572346
*Microsphaeropsiscytisina*	*Neomicrosphaeropsiscytisina*	MFLU 16-1364	T	*Cytisusscoparius*	Italy	KX611243	KX611241			KX611242
*Microsphaeropsiscystisicola*	*Neomicrosphaeropsiscystisicola*	MFLUCC 18-0355	T	*Cytisus* sp.	Italy	MH069665	MH069671	MH069690	−	−
*Microsphaeropsiscytisi*	*Neomicrosphaeropsiscystisi*	MFLUCC 13-0396	T	*Cytisus* sp.	Italy	KX572337	KX572342	−	−	KX572347
	*Microsphaeropsisfusca*	CBS 116670	T	Sarothamnus scoparius	The Netherlands	MN973573	MT018220	−	MT018220	−
	*Microsphaeropsisrafniae*	CMW 57792	T	Rafnia amplexicaulis	South Africa	OR209698	OR209716	−	OR211858	−
	*Microsphaeropsisviridis*	CBS 763.73	F	Populus tremula	France	MN973561	MN943768	−	MT018210	−
	*Microsphaeropsistaxicola*	CBS 469.80	F	Rhus typhina	The Netherlands	MN973565	MN943772	−	MT018210	−
	*Neodidymelliopsisranunculi*	MFLUCC 13-0490	T	Unidentified	Italy	MN944410	MT020377	−	−	KX572348
	*Neoascochytaadenii*	CBS 142108	T	*Adeniumobesum*	Thailand	KY173423	KY173514	KY173607	−	−
	*Neoascochytaargentina*	CBS 112524	T	*Triticumaestivum*	Argentina	KT389524	KT389742	KT389822	−	−
	*Neoascochytacylindrispora*	CBS 142456	T	*Homosapiens*	USA	LT592963	LN907502	LT593032	−	−
	*Neoascochytadactylidis*	MFLUCC 13-0495	T	*Dactylisglomerata*	Italy	NR_170041	−	−	−	−
	*Neoascochytadesmazieri*	CBS 297.69	T	*Loliumperenne*	Germany	KT389508	KT389726	KT389806	−	−
	*Neoascochytadesmazieri*	CBS 758.97	F	Unidentified	Norway	KT389509	KT389727	KT389807	−	−
	*Neoascochytadesmazieri*	CBS 247.79	F	Gramineae	Austria	KT389507	KT389725	KT389805	−	−
	*Neoascochytaeuropaea*	CBS 820.84	T	*Hordeumvulgare*	Germany	KT389511	KT389729	KT389809	−	−
	*Neoascochytaeuropaea*	CBS 819.84	F	*Hordeumvulgare*	Germany	KT389510	KT389728	KT389808	−	−
	*Neoascochytaexitialis*	CBS 812.84	F	*Hordeumvulgare*	Germany	KT389517	KT389735	KT389815	−	−
	*Neoascochytaexitialis*	CBS 811.84	F	*Secalecereale*	Germany	KT389516	KT389734	KT389814	−	−
	*Neoascochytaexitialis*	CBS 389.86	F	*Triticumaestivum*	Switzerland	KT389515	KT389733	KT389813	−	−
	*Neoascochytaexitialis*	CBS 113693	F	*Allium* sp.	Sweden	KT389513	KT389731	KT389811	−	−
	*Neoascochytaexitialis*	CBS 110124	F	*Triticum* sp.	Netherlands	KT389512	KT389730	KT389810	−	−
	*Neoascochytafuci*	CMG 47/MUM19.41	T	*Fucus* sp.	Portugal	MN053014	−	MN066618	−	−
	*Neoascochytafuci*	CMG 48	F	*Fucus* sp.	Portugal	MN053015	−	MN066619	−	−
	*Neoascochytafusiformis*	CBS 876.72	T	*Triticum* sp.	South Africa	KT389527	KT389745	KT389825	−	−
	*Neoascochytagraminicola*	CBS 816.84	F	*Hordeumvulgare*	Germany	KT389523	KT389741	KT389821	−	−
	*Neoascochytagraminicola*	CBS 815.84	F	*Hordeumvulgare*	Germany	KT389522	KT389740	KT389820	−	−
	*Neoascochytagraminicola*	CBS 447.82	F	*Triticumaestivum*	Germany	KT389520	KT389738	KT389818	−	−
	*Neoascochytagraminicola*	CBS 301.69	F	*Loliummultiflorum*	Germany	KT389519	KT389737	KT389817	−	−
	*Neoascochytagraminicola*	CBS 102789	F	*Loliumperenne*	New Zealand	KT389518	KT389736	KT389816	−	−
	*Neoascochytahumicola*	CBS 127323	T	Unidentified	USA	MN973628	MN943837	MT005740	−	−
	*Neoascochytalongispora*	CBS 113420	T	*Cerastiumsemidecandrum*	New Zealand	MN973629	MN943838	MT005741	−	−
	*Neoascochytamortariensis*	CBS 516.81	T	Gramineae	Italy	KT389525	KT389743	KT389823	−	−
	*Neoascochytapaspali*	CBS 560.81	T	*Paspalumdilatatum*	New Zealand	FJ427048	GU238124	FJ427158	−	−
	*Neoascochytapaspali*	CBS 561.81	F	*Loliumperenne*	New Zealand	GU237889	−	GU237640	−	−
	*Neoascochytapaspali*	ICMP 6614	F	*Paspalumdilatatum*	New Zealand	KT309957	−	KT309539	−	−
	*Neoascochytapaspali*	ICMP 6819	F	*Dactylisglomerata*	New Zealand	KT309992	−	KT309572	−	−
	*Neoascochytapaspali*	ICMP 6615	F	*Loliumperenne*	New Zealand	KT309958	−	KT309540	−	−
	*Neoascochytarosicola*	MFLUCC 15-0048	T	*Rosacanin*a	Italy	MG828921	MG829031	−	−	−
	*Neoascochytasoli*	LC 8165	T	Unidentified	China	KY742121	KY742275	KY742363	−	−
	*Neoascochytasoli*	LC 8166	F	Unidentified	China	KY742122	KY742276	KY742364	−	−
	*Neoascochytatardicrescens*	CBS 689.97	T	Unidentified	Norway	KT389526	KT389744	KT389824	−	−
	*Neoascochytatriticicola*	CBS 544.74	T	*Triticumaestivum*	South Africa	GU237887	EU754134	GU237488	−	−
	*Neoascochytayunnanensis*	YCW1883	T	*Camelliasinensis*	China	OP648090	OP837280	OP854553		
	*Neoascochytazhejiangensis*	YCW1361	T	*Camelliasinensis*	China	OP648091	OP083837281	OP854554		
	***Neoascochytapseudofusiformis***	**GUCC 23-0045**	**T**	**Unidentified**	**China**	**PP314026**	**PP314037**	**PP396053**	**PP396047**	**PP345789**
	***Neoascochytapseudofusiformis***	**GUCC 23-0046**	**F**	**Unidentified**	**China**	**PP314027**	**PP314038**	**PP396054**	**PP396048**	**PP301319**
	***Neoascochytapseudofusiformis***	**GUCC 23-0047**	**F**	**Unidentified**	**China**	**PP314029**	**PP314036**	**PP396055**	**PP396049**	**PP301320**
	*Vandijckomycellajoseae*	CBS 143011	T	Unidentified	Unknown	NR_168247	NG_068687	−	−	−
	*Vandijckomycellasnoekiae*	CBS 144954	T	Unidentified	Unknown	NR_168248	NG_068688	MN824765	−	−

### ﻿Sequence alignment and phylogenetic analyses

**Figure 1.**
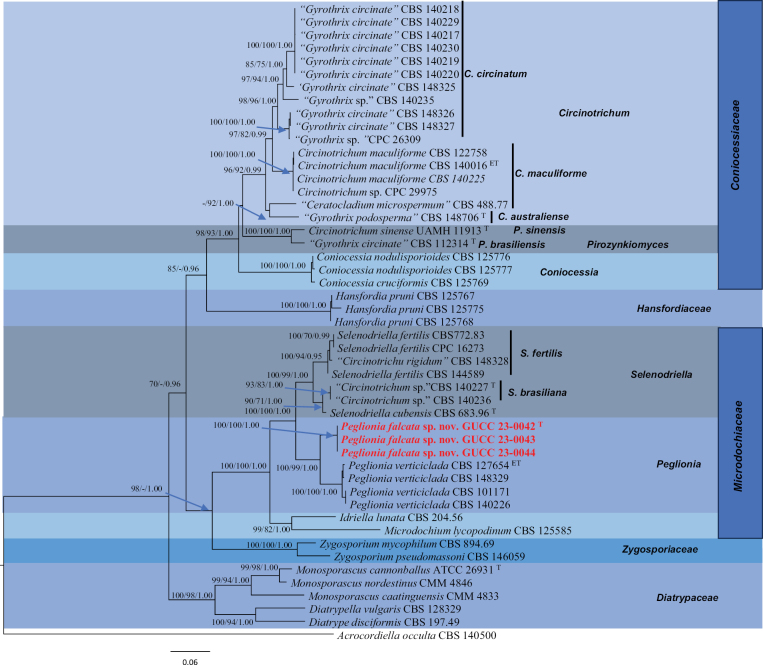
Trees resulting from ML analysis of the combined ITS, *rpb*2 and LSU sequence alignment for forty-nine isolates in Coniocessiaceae and Microdochiaceae. RAxML and MP bootstrap support values (ML, MP ≥ 70%) and Bayesian posterior probability (PP ≥ 0.95) are denoted on the nodes (ML/MP/PP). The tree was rooted to *Acrocordiellaocculta* (CBS 140500). New species are highlighted in red. The scale bar indicates 0.06 expected changes per site. T = ex-holotype strain, ET = ex-epitype strain.

**Figure 2.**
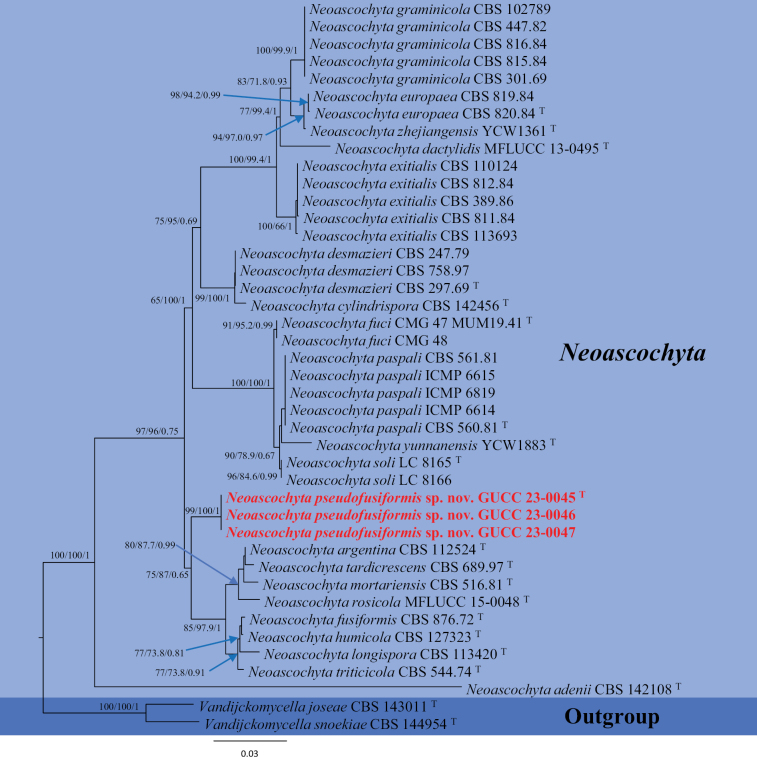
Trees resulting from ML analysis of the combined ITS, *tub2* and LSU sequence alignment for thirty-seven isolates in *Neoascochyta*. RAxML and MP bootstrap support values (ML, MP ≥ 65%) and Bayesian posterior probability (PP ≥ 0.65) are denoted on the nodes (ML/MP/PP). The tree was rooted to *Vandijckomycellajoseae* (CBS 143011) and *Vandijckomycellasnoekiae* (CBS 144954). New species are highlighted in red. The scale bar indicates 0.03 expected changes per site. T = ex-holotype strain.

**Figure 3.**
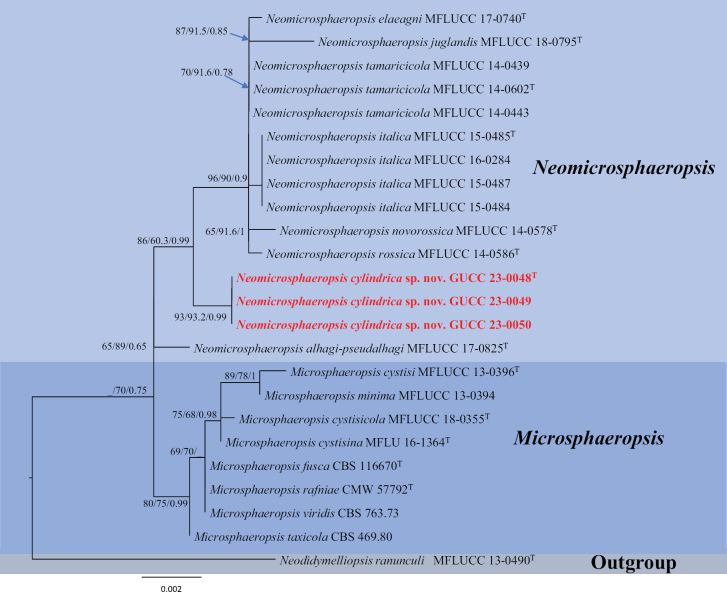
Trees resulting from ML analysis of the combined ITS, SSU and LSU sequence alignment for twelve isolates in *Neomicrosphaeropsis* and eight isolates in *Microsphaeropsis*. RAxML and MP bootstrap support values (ML, MP ≥ 60%) and Bayesian posterior probability (PP ≥ 0.65) are denoted on the nodes (ML/MP/PP). The tree was rooted to *Neodidymelliopsisranunculi* (MFLUCC13-0490). New species are highlighted in red. The scale bar indicates 0.002 expected changes per site. T = ex-holotype strain.

After primary BLAST alignment, all our nine isolates could not be affiliated to any of the currently-known species. Thus, the related sequences were added to the sequence alignment for phylogenetic analyses. Available sequences of species in relative genera containing ex-type or representative isolates were downloaded from GenBank (Table 1) according to previous publications (Li et al. 2018, 2020; Vu et al. 2019; Chen et al. 2020; Chu et al. 2021; Yukako et al. 2021). Alignments for the individual locus matrices were generated with the online version of MAFFT v. 7.307 (Katoh et al. 2019). The alignments were checked visually and improved manually where necessary using BioEdit v. 7.0.5.2 (Hall 1999). Ambiguous regions were excluded from the analyses and gaps were treated as missing data. Sequence matrix v. 1.7.8 was used to concatenate the aligned sequences (Vaidya et al. 2011). In Fig. 1, *Acrocordiellaocculta* (CBS 140500) was selected as outgroup, in Fig. 2, *Vandijckomycellajoseae* (CBS 143011) and *V.snoekiae* (CBS 144954) were selected as outgroup and, in Fig. 3, *Neodidymelliopsisranunculi* (MFLUCC 13-0490) was selected as outgroup. Maximum Likelihood (ML), Maximum Parsimony (MP) and Bayesian Inference (BI) were used to place the newly-discovered strains into a phylogenetic framework and estimate their phylogenetic relationships. ML analysis was performed using IQ-TREE (Nguyen et al. 2015; Trifinopoulos et al. 2016) on the IQ-TREE web server (http://iqtree.cibiv.univie.ac.at, 17 February 2024). The MP analysis was implemented to test the discrepancy of the ITS, *rpb2*, LSU, SSU and *tub2* sequence datasets with PAUP v. 4.0b10 (Swofford 2002). Gaps were treated as missing data, which were interpreted as uncertainty of multistate taxa. MP trees were generated using the heuristic search option with tree bisection re-connection (TBR) branch swapping. “Maxtrees” was set to 5000, the tree length (TL), consistency index (CI), homoplasy index (HI), retention index (RI) and rescaled consistency index (RC) were calculated. BI was performed using six Markov Chain Monte Carlo runs for 5,000,000 generations, sampling every 1000 generations. The first 25% resulting trees were discarded as the burn-in phase of each analysis.

## ﻿Results

In the phylogenetic analyses, the MP, ML and Bayesian results obtained similar topologies, thus the ML topologies were edited and shown as Figs 1–3. For the *Peglionia* and related genera (Fig. 1), the combined data matrix of ITS–LSU–*rpb*2 consisted of 2366 characters (ITS: 761, LSU: 885 and *rpb*2: 720), amongst which 612 are parsimony informative characters. Maximum Parsimony analysis with the following parameters: TL = 2197; CI = 0.5294; HI = 0.4706; RI = 0.8182; and RC = 0.4331 indicated that *Peglioniafalcata* strains (GUCC-0042, GUCC-0043 and GUCC-0044) without the DNA base differences in three loci formed an independent branch (ML = 100, MP = 100, PP = 1.00) and maintained a close relationship to *P.verticiclada* (CBS 127654, CBS 148329, CBS 101171 and CBS 140226) (ML = 100, MP = 99, PP = 1.00).

The combined data matrix of *Neoascochyta* (ITS–LSU–*tub*2) yielded 1784 characters (ITS: 489, LSU: 959 and *tub*2: 336). The MP analysis, based on 194 parsimony informative characters (1480 characters were constant and 110 variable characters), produced the phylogenetic tree with the following parameters: TL = 562; CI = 0.6975; HI = 0.3025; RI = 0.8932; and RC = 0.6230. The result (Fig. 2) displayed that *Neoascochytapseudofusiformis* (GUCC23-0045, GUCC23-0046 and GUCC23-0047) formed an independent branch without the DNA base differences in three loci supported by strong statistic data (ML = 99, MP = 100, PP = 1.00) and were adjacent to the branch of *Neoascochytaargentina* CBS 112524 and *N.tardicrescens* (CBS 689.97) (ML = 75, MP = 87, PP = 0.65).

In Fig. 3, the combined data matrix of *Neomicrosphaeropsis* (ITS–LSU–SSU) including 2328 characters (ITS: 484, LSU: 833 and SSU: 1011), only had 17 parsimony informative characters. The MP analysis (TL = 51; CI = 0.8627; HI = 0.1373; RI = 0.9136; and RC = 0.7882) indicated the three strains of *Neomicrosphaeropsiscylindrica* (GUCC 23-0048, GUCC 23-0049 and GUCC 23-0050) as a branch without genetic distance (ML = 93, MP = 93.2, PP = 0.99) adjoining to the clade (ML = 86, MP = 60.3, PP = 0.99) including *Neom.rossica*, *Neom.novorossica* and *Neom.italica*. The phylogenetic placements of these novel taxa were also supported by DNA base-pair differences (Table 2).

**Table 2.** The DNA base differences of our isolates and related taxa in different loci.

Species	Strain number	ITS (1–761 characters)	LSU (762–1646 characters)	*rpb*2 (1647–2366 characters)
***Peglioniafalcata***	GUCC-0042	0	0	0
***Peglioniafalcata***	GUCC-0043	0	0	0
***Peglioniafalcata***	GUCC-0044	0	0	0
*Peglioniaverticiclada*	CBS 127654	20 (gaps: 4)	13 (gap: 0)	69 (gap: 0)
*Peglioniaverticiclada*	CBS 101171	19 (gaps: 4)	17 (gap: 1)	65 (gap: 0)
*Peglioniaverticiclada*	CBS 683.96	36 (gaps: 7)	25 (gaps: 2)	/
*Peglioniaverticiclada*	CBS 140227	39 (gaps: 6)	26 (gaps: 2)	84 (gap: 0)
**Species**	**Strain number**	**ITS (1–489 characters)**	**LSU (490–1448 characters)**	***tub*2 (1449–1784 characters)**
***Neoascochytapseudofusiformis***	GUCC23-0045	0	0	0
***Neoascochytapseudofusiformis***	GUCC23-0046	0	0	0
***Neoascochytapseudofusiformis***	GUCC23-0047	0	0	0
*Neoascochytasoli*	LC 8166	24 (gap: 3)	16 (gap: 0)	35 (gap: 1)
*Neoascochytaargentina*	CBS 112524	18 (gap: 0)	2 (gap: 0)	29 (gap: 1)
*Neoascochytatardicrescens*	CBS 689.97	23 (gap: 0)	2 (gap: 0)	30 (gap: 1)
*Neoascochytamortariensis*	CBS 516.81	20 (gap: 0)	2 (gap: 0)	30 (gap: 1)
*Neoascochytarosicola*	MFLUCC 15-0048	24 (gaps: 0)	3 (gap: 0)	/
**Species**	**Strain number**	**ITS (1–484 characters)**	**LSU (485–1317)**	**SSU (1318–2328 characters)**
***Neomicrosphaeropsiscylindrica***	GUCC 23-0048	0	0	0
***Neomicrosphaeropsiscylindrica***	GUCC 23-0049	0	0	0
***Neomicrosphaeropsiscylindrica***	GUCC 23-0050	0	0	0
*Neomicrosphaeropsisrossica*	MFLUCC 14-0586	4 (gap:0)	4 (gap: 0)	1 (gap: 1)
*Neomicrosphaeropsisnovorossica*	MFLUCC 14-0578	5 (gap:0)	3 (gap: 0)	1 (gap: 0)
*Neomicrosphaeropsisalhagi-pseudalhagi*	MFLUCC 17-0825	5 (gaps:0)	2 (gap: 0)	0

### ﻿Taxonomy

#### ﻿ Peglionia falcata

Taxon classificationFungiXylarialesMicrodochiaceae

S.M. Fu & Yong Wang bis sp. nov.

974A76F0-EFC1-50B7-AC9C-FF1C601CA005

854204

Facesoffungi Number: FoF15890

Fig. 4

##### Etymology.

In reference to the fungus, which produced falcate conidia.

##### Diagnosis.

*Peglioniafalcata* is characterised by dry falcate meriform spores (24.1 × 2.9 μm; L/W = 8.005).

**Figure 4.**
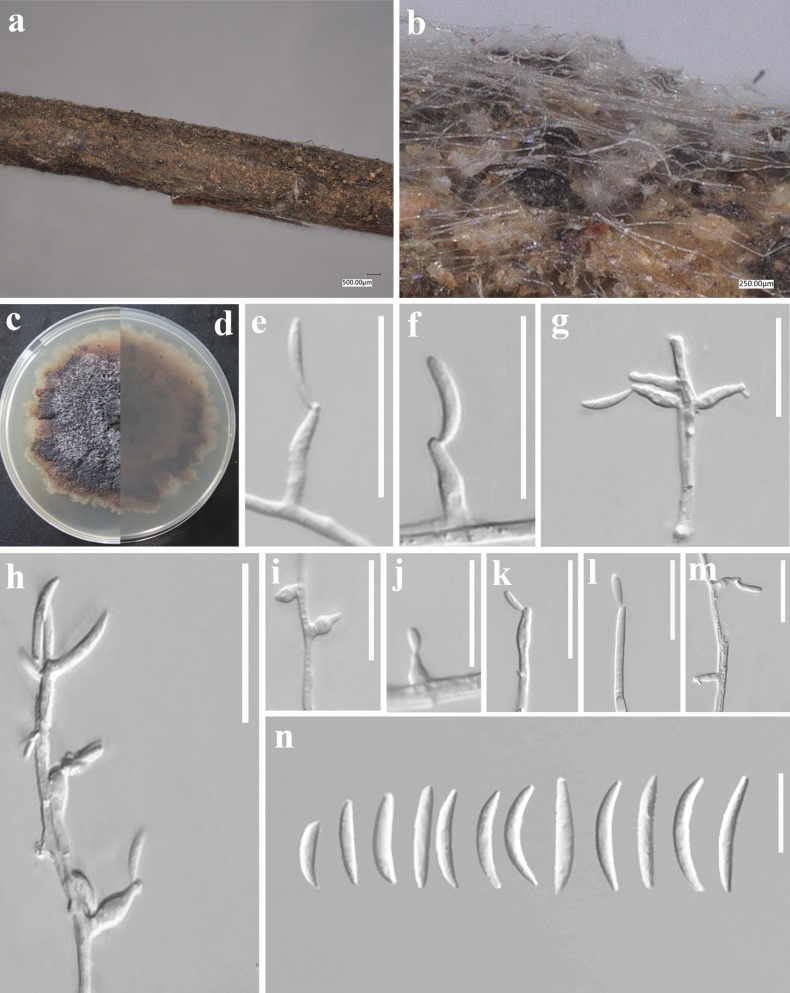
*Peglioniafalcata* (GUCC23-0042) **a, b** appearance on host surface **c, d** culture characteristics on PDA (**c** above view **d** reverse view) **e–m** conidiophores, conidiogenous cells and conidia **n** conidia. Scale bars: 20 µm (**e–n**).

##### Type.

China, Guizhou Province, Guiyang City, 26°57'N, 106°72'E, from rotten dead branch, 19 July 2023, S.M. Fu, HGUP 23-0013 (holotype), ex-type culture GUCC23-0042.

##### Culture characteristics.

Colonies on PDA, after 8 d, 20–25 mm diam., scarce aerial mycelium, dark brown, white to the periphery, margin entire, reverse dark brown. Occasionally, when a seta bears only two apical branches, one or both can be once forked. Conidiogenous cells polyblastic, obclavate to lageniform, hyaline to subhyaline. Conidia adherent in a continuous white layer on the conidiogenous cells, dry falcate, non-septate, hyaline. Chlamydospores (in culture) in chains, subglobose to irregularly-shaped, subhyaline to brown. Sexual morph not observed. Colonies hypophyllous, scattered, up to 1 mm wide, occasionally larger by confluence, velvety, black when sterile and whitish within when sporulating profusely. Conidiogenous cells obclavate to lageniform, hyaline to subhyaline, distally with a somewhat irregular contour, 5–16.5 × 4–7 µm (x̄ = 9.8 × 5.3 μm, n = 20). Conidia adherent in a continuous white layer on the conidiogenous cells, falcate, non-septate, hyaline,18–30 × 2.5–3.5 µm (x̄ = 24.1 × 2.9 μm, n = 30).

##### Habit.

On rotten dead branches.

##### Distribution.

China, Guizhou Province, Guiyang City

##### Other materials examined.

China, Guizhou Province, Guiyang City, 26°57'N, 106°72'E, from rotten dead branch, 19 July 2023, S.M. Fu, HGUP 23-0013, living culture GUCC23-0042, GUCC23-0043 and GUCC23-0044.

##### Notes.

In morphology, *Peglioniafalcata* differs to *P.verticiclada* by its larger conidiogenous cells (4–7 μm wide vs. 3–5 μm wide in *P.verticiclada*) and larger conidia (18–30 μm vs. 17–22 μm in *P.verticiclada*) (Hernández-Restrepo et al. 2022). The phylogenetic analyses and DNA base differences (Table 2) also supported *P.falcata* as a novel taxon was distinct from *P.verticiclada*.

#### ﻿ Neoascochyta pseudofusiformis

Taxon classificationFungiPleosporalesDidymellaceae

S.M. Fu & Yong Wang bis sp. nov.

0E7A0112-977D-553E-8AA8-1C70D7C6D535

854206

Facesoffungi Number: FoF15891

Fig. 5

##### Etymology.

In reference to the fungus, which produced fusiform conidia morphologically similar to *Neoascochytafusiformis*.

**Figure 5.**
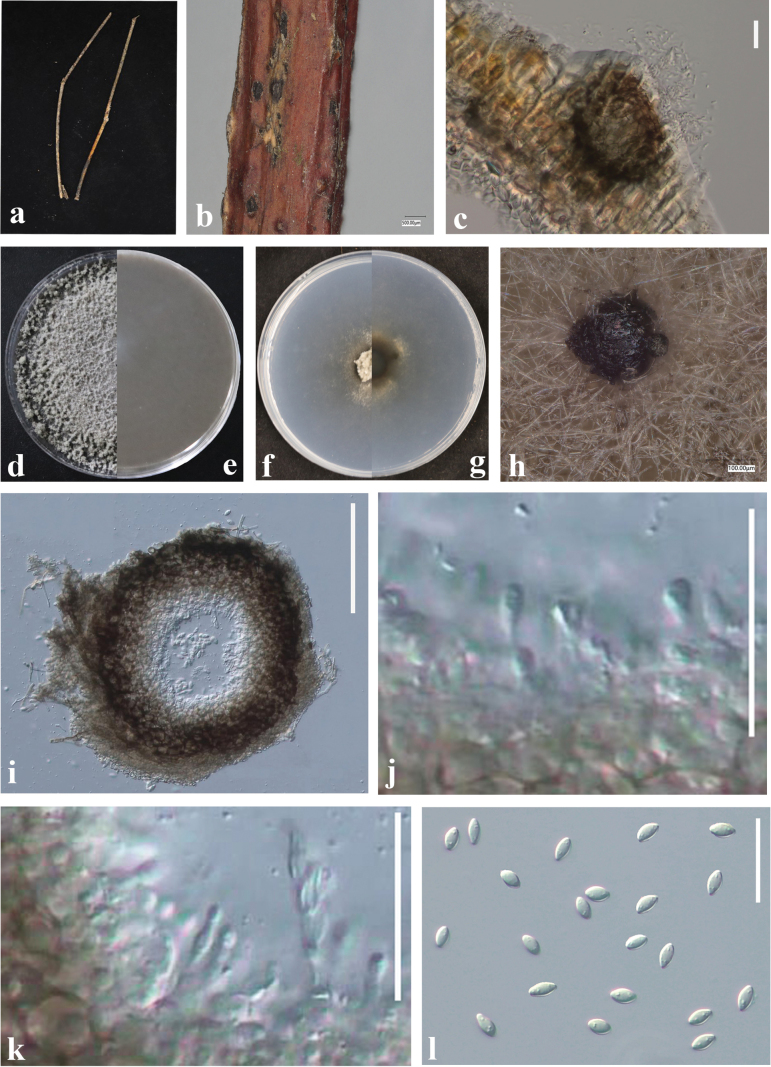
*Neoascochytapseudofusiformis* (GUCC 23-0045) **a, b** appearance on host surface **c** colonies on host surface **d–g** culture characteristics on PDA, WA (**d, f** above view **e, g** reverse view) **h** colonies on WA **i–k** conidiophores, conidiogenous cells and conidia **l** conidia. Scale bars: 20 µm (**i**–**l**).

##### Diagnosis.

*Neoascochytapseudofusiformis* is characterised by oval to fusiform conidia (3.6 × 1.9; L/W = 1.896) with moderate growth rate.

##### Type.

China, Guizhou Province, Guiyang City, 26°57'N, 106°72'E, from rotten dead branch, 19 July 2023, S.M. Fu, HGUP 23-0014 (holotype), ex-type culture GUCC23-0045.

##### Culture characteristics.

Colonies on PDA, 70–75 mm diam. after 7 d, margin regular, covered by floccose aerial mycelium, greyish-olivaceous, with flat and greenish-black flat mycelium near the margin; reverse black olivaceous. Mycelium is light to dark grey, separated, smooth, thin to thick wall. Acicular conidium, grey to dark grey, solitary or conjunctival, immersed in culture (WA), glabrous, subglobular, 100–250 × 90–130 μm, with a single pore neck; The angular textured cylindrical wall consists of 2 to 4 layers of flat polygonal cells 10–50 μm thick. The meristem cells are biparental, transparent, smooth-walled, pot or spherical, 3 × 5 μm wide. The conidia are 0–1 septum, transparent, smooth, thick-walled, mostly fusiform or slightly allantoic, 3.0–4.5 × 1.5–2.5 μm (x̄ = 3.6 × 1.9 μm, n = 30).

##### Habit.

On rotten dead branches.

##### Distribution.

China, Guizhou Province, Guiyang City

##### Other materials examined.

China, Guizhou Province, Guiyang City, 26°57'N, 106°72'E, from rotten dead branches, 19 July 2023, S.M. Fu, HGUP 23-0014, living culture GUCC23-0045, GUCC23-0046 and GUCC23-0047.

##### Notes.

The present taxon differs morphologically to related species by conidial size range (3.0–4.5 × 1.5–2.5 μm vs. 16.5–27 × 5–8.5 μm in *N.argentina* and 2.5–3.5 × 1.0–1.5 μm in *N.tardicrescens*) (Chen et al. 2017; Valenzuela-Lopez et al. 2018). In phylogeny, our novel taxon maintained a close relationship to *N.argentina* CBS 112524 and *N.tardicrescens* CBS 689.97; however, DNA base differences (Table 2) supported that they belonged to different taxa.

#### ﻿ Neomicrosphaeropsis cylindrica

Taxon classificationFungiPleosporalesDidymellaceae

S.M. Fu & Yong Wang bis sp. nov.

E8432987-2441-5A17-8ECA-566C20359DD3

854207

Facesoffungi Number: FoF15892

Fig. 6

##### Etymology.

In reference to the fungus, which produced cylindrical conidia.

##### Diagnosis.

*Neomicrosphaeropsiscylindrica* is characterised by broadly cylindrical conidia (15.4 × 3.4; L/W = 4.574) with moderate growth rate.

**Figure 6.**
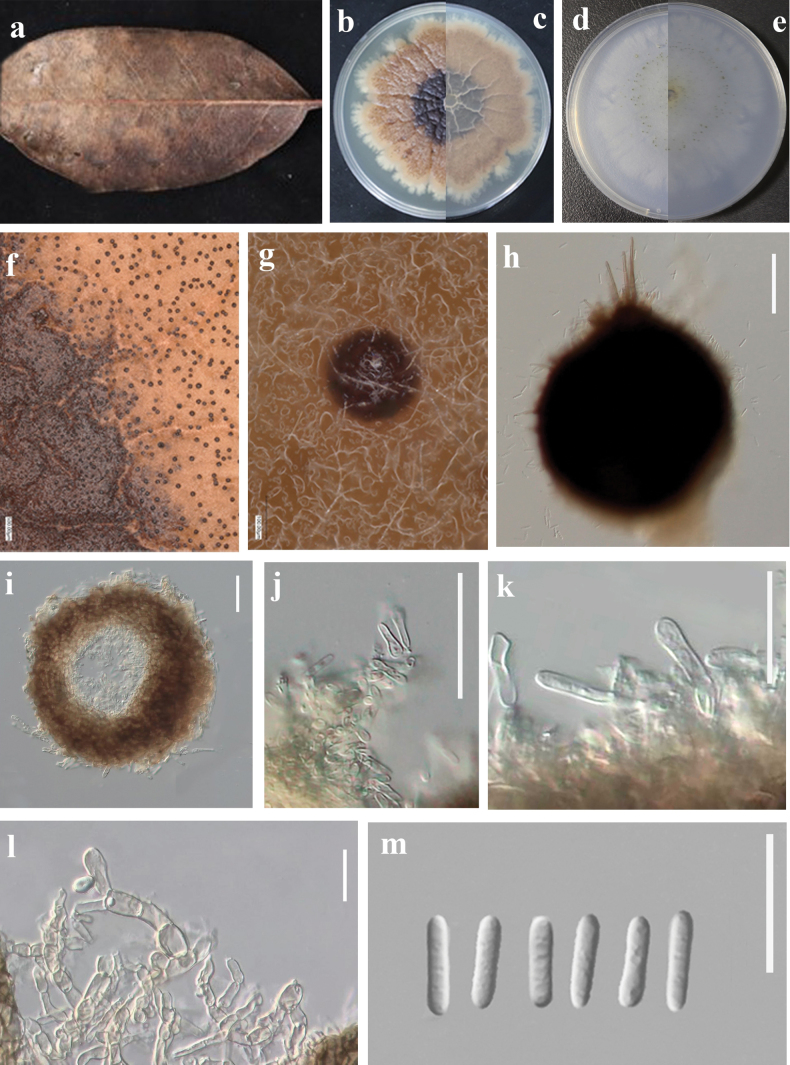
*Neomicrosphaeropsiscylindrica* (GUCC 23-0048) **a** appearance on host surface **b–e** culture characteristics on PDA, WA (**b–d** above view **c–e** reverse view) **f–g** colonies on PDA**h–l** conidiophores, Conidiogenous cells and Conidia **m** conidia. Scale bars: 100 µm (**h**); 50 µm (**i–k**); 20 µm (**l–m**).

##### Type.

China, Guizhou Province, Guiyang City, 26°57'N, 106°72'E, from rotten dead leaves, 19 July 2023, S.M. Fu, HGUP 23-0015 (holotype), ex-type culture GUCC23-0048.

##### Culture characteristics.

Saprobic on dead leaves. Colony on PDA, 35–38 mm diameter, after 7 days, dense low-altitude hypha, light yellow, centre with abundant stigma; Turning light yellow to rose light yellow, the centre of concentric circles is darker; on MEA, after 7 days, 28–30 mm, the edge is intact, dense hypoxic mycelium, the edge is yellowish; reverse rose-yellowish to yellowish at margin with abundant scattered on stigma; Conidia cylindrical, spherical to kettle-shaped, 200–350 μm in diameter, tan to black, solitary, population centre abundant, banded, glabrous, without papillae; the cell wall is angular textured, light brown, bifid, cylindrical, thin-walled, transparent. Conidia occasionally septate, 12.5–18.5 × 2.4–4.0 μm (x̄ = 15.4 × 3.4 μm, n = 30), cylindrical, transparent, thin-walled.

##### Sexual stage.

Not observed.

##### Habit.

On rotten dead leaves.

##### Distribution.

China, Guizhou Province, Guiyang City.

##### Other materials examined.

China, Guizhou Province, Guiyang City, 26°57'N, 106°72'E, from rotten dead leaf, 19 July 2023, S.M. Fu, HGUP 23-0015, living culture GUCC23-0048, GUCC23-0049 and GUCC23-0050.

##### Notes.

*Neomicrosphaeropsiscylindrica* (GUCC 23-0048) formed a clade with *N.rossica* (MFLUCC 14-0586) and *N.alhagi-pseudalhagi* (MFLUCC 17-0825) (Fig. 3). However, by morphological comparison, our species produced obviously longer conidia than *N.rossica* (12.5–18.5 × 2.4–4.0 μm vs. 4.4–5.7 × 2.9–3.9 μm) and smaller conidia than *N.alhagi-pseudalhagi* (12.5–18.5 × 2.4–4.0 μm vs. 30–45 × 18–22 μm) (Thambugala et al. 2017; Wanasinghe 2018).

## ﻿Discussion

According to Hernández-Restrepo et al. (2022), four strains of *Peglioniaverticiclada* totally originated from decayed plant tissues in Europe and Australia. Our *Peglionia* species was also collected from rotten dead branches, which was the first discovery in China. In the database of Index Fungorum, *Neoascochyta* has 20 epithets and three of them were described by Chinese mycologists (Chen et al. 2017; Wang et al. 2024). Interestingly, the plant hosts of *Neoascochyta* spp. were mostly reported in the Gramineae family, such as *Triticum* sp., *Hordeum* sp. and *Paspalum* sp. (Chen et al. 2015, 2017; Hou et al. 2020). However, only two taxa (*Neoascochytasoli* and our *N.pseudofusiformis*) were both isolated from Guizhou, China and related to saprobic environments (Chen et al. 2017). In the test of inhibiting the germination of rust spores, the number of rust spots on leaves were significantly reduced after *Neoascochyta* treatment, which may provide a potential biological control method against rust diseases (Wilson et al. 2020). *Neomicrosphaeropsiscylindrica* was also the first species in *Neomicrosphaeropsis* to be discovered and described in China. This genus presented high correlations with alcohol and acids and was the highest contributors to the generation of volatile compounds, especially during alcohol production (Ma et al. 2021). Members of this genus were mostly obtained from Salicaceae as saprophytic fungi (Hyde et al. 2016; Thambugala et al. 2017). All our three Pezizomycotina taxa were isolated from rotten branches or leaves, which indicated that the diversity of this fungal group in Guizhou was relatively high. Thus, there was a need for a more comprehensive investigation.

## Supplementary Material

XML Treatment for Peglionia falcata

XML Treatment for Neoascochyta pseudofusiformis

XML Treatment for Neomicrosphaeropsis cylindrica

## ﻿Additional information

### Author ORCIDs

Shamin Fu https://orcid.org/0009-0000-2829-7967

Jing-E Sun https://orcid.org/0000-0002-5226-5743

Entaj Tarafder https://orcid.org/0000-0002-3680-3433

Nalin N. Wijayawardene https://orcid.org/0000-0003-0522-5498

Yong Wang https://orcid.org/0000-0003-3831-2117

Yan Li https://orcid.org/0000-0003-2227-008X

### Data availability

All of the data that support the findings of this study are available in the main text.
